# Single-cell intracellular pH dynamics regulate the cell cycle by timing the G1 exit and G2 transition

**DOI:** 10.1242/jcs.260458

**Published:** 2023-05-31

**Authors:** Julia S. Spear, Katharine A. White

**Affiliations:** ^1^ Department of Chemistry and Biochemistry, University of Notre Dame, Notre Dame, IN 46556, USA; ^2^ Harper Cancer Research Institute, University of Notre Dame, South Bend, IN 46617, USA

**Keywords:** Cell cycle, Mitosis, Intracellular pH, pH biosensor, Single-cell methods, Quantitative imaging

## Abstract

Transient changes in intracellular pH (pHi) regulate normal cell behaviors, but roles for spatiotemporal pHi dynamics in single-cell behaviors remain unclear. Here, we mapped single-cell spatiotemporal pHi dynamics during mammalian cell cycle progression both with and without cell cycle synchronization. We found that single-cell pHi is dynamic throughout the cell cycle: pHi decreases at G1/S, increases in mid-S, decreases at late S, increases at G2/M and rapidly decreases during mitosis. Importantly, although pHi is highly dynamic in dividing cells, non-dividing cells have attenuated pHi dynamics. Using two independent pHi manipulation methods, we found that low pHi inhibits completion of S phase whereas high pHi promotes both S/G2 and G2/M transitions. Our data also suggest that low pHi cues G1 exit, with decreased pHi shortening G1 and increased pHi elongating G1. Furthermore, dynamic pHi is required for S phase timing, as high pHi elongates S phase and low pHi inhibits S/G2 transition. This work reveals that spatiotemporal pHi dynamics are necessary for cell cycle progression at multiple phase transitions in single human cells.

## Teaser

**Highlighted Article:** Single-cell intracellular pH (pHi) measurements reveal oscillations during the mammalian cell cycle. Manipulating pHi alters phase lengths and transitions, suggesting pHi dynamics regulate the cell cycle.

## INTRODUCTION

In normal epithelial cells, intracellular pH (pHi) is near neutral (∼7.2) whereas extracellular pH (pHe) is more alkaline (∼7.4) (White et al., 2017a). Transient changes in pHi driven by ion transporter activity (Boron, 2004) have been shown to regulate normal cell behaviors, such as differentiation (Ulmschneider et al., 2016), proliferation (Flinck et al., 2018b), migration (Choi et al., 2013; Martin et al., 2011) and apoptosis (Sergeeva et al., 2017). However, most studies of pHi-dependent cell behaviors are limited because average pHi is measured across a population of cells, pHi measurements are performed in non-native cellular environments, or pHi is monitored over short timeframes during a long biological process. Thus, our understanding of how spatiotemporal single-cell pHi dynamics regulate cell behaviors is limited. Better understanding of how pHi dynamics drive single-cell behaviors will reveal mechanistic roles for pHi in regulating biology and validate pHi as a reporter of cell phenotype.

One pHi-dependent behavior where rigorous spatiotemporal single-cell pHi measurements could help reveal mechanism is cellular proliferation. Links between cell cycle and pHi were first identified in unicellular organisms, such as tetrahymena (Gillies and Deamer, 1979), *Dictyostelium* (Aerts et al., 1985) and *Schizosaccharomyces pombe* (Karagiannis and Young, 2001). In tetrahymena, two increases in pHi (∼0.4 pH units) were observed pre- and post-S phase (Gillies and Deamer, 1979). However, all measurements of pHi changes were made at the population level and used harsh synchronization techniques (starvation and heat shock), which can disrupt essential cell metabolic functions in addition to regulating the cell cycle (Gillies and Deamer, 1979). In *Dictyostelium*, increased pHi (∼0.2 pH units) was measured during S phase, and when pHi was artificially increased, DNA replication and protein synthesis were increased (Aerts et al., 1985). However, no timing or delays in S phase progression were noted with pHi manipulation, and only population-level pHi measurements were reported. In conflict with these two previous studies, no relationship between cell cycle progression and pHi was found in *S. pombe* when pHi was monitored using a genetically encoded pHi biosensor (pHluorin) (Karagiannis and Young, 2001). Therefore, the existing data in unicellular organisms is inconsistent on whether pHi dynamics are sufficient to regulate (or time) cell cycle progression. We note that these inconsistencies could be biologically meaningful (due to species-specific differences in cell cycle regulation) or the inconsistencies could be artifactual (due to non-physiological pHi measurements and manipulations in these models).

Some studies in animal cell models have also shown a relationship between pH and cell cycle progression. In quiescent populations of human tumor cells, it has been shown that a narrow range of pHe values (pH 6.8 to 7.2) are required to recruit cells into the cell cycle (Taylor and Hodson, 1984). Although this suggests that a defined range of pHe is required for normal proliferation, the authors did not measure pHi during these experiments (Taylor and Hodson, 1984). Population-level analyses of pHi in thymidine-synchronized MCF-7 breast cancer cells showed that pHi fluctuated after thymidine release but no statistical significance was noted (Flinck et al., 2018a). Strengthening the link between pHi and cell cycle regulation, knockdown of the Na^+^-H^+^ exchanger (NHE1) and the Na^+^-HCO_3_^−^ transporter (NBCn1) causes elongation of S phase and a delay in the G2/M transition in breast cancer cells (Flinck et al., 2018a), but single-cell pHi was not measured. In another example, an increase in pHi driven by NHE1 was found to be required for G2/M transition in fibroblasts, but single-cell pHi was not measured, and pH was manipulated using genetic knockout or overexpression of NHE1 (Putney and Barber, 2003). As ion transporters also serve scaffolding and signaling roles, genetic knockdown produces transport-independent effects on cell biology. In summary, although these studies lay a strong framework for a relationship between pHi and the cell cycle, single-cell pHi measurements in mammalian cells cultured under native environments are needed to elucidate how temporal pHi dynamics regulate cell cycle progression.

Here, we measure single-cell pHi under physiological growth conditions in both asynchronous and synchronized human lung-derived cell populations to determine how pHi regulates cell cycle progression in single cells. We found that single-cell pHi oscillates during cell cycle progression. Importantly, we determined that pHi oscillations correlate with cell cycle stages: pHi decreases near the G1/S transition, increases during mid-S, decreases again at S/G2 transitions, and finally increases at G2/M followed by rapid acidification during mitosis. Using pHi manipulation and fluorescent ubiquitylation-based cell cycle indicator (FUCCI) reporters, we determined that dynamic pHi is necessary for normal cell cycle progression. Similar to what was found in prior work, we show that increased pHi is required for successful completion of G2/M. But our work also reveals previously uncharacterized pHi dynamics regulating both G1 exit and S phase duration. This work highlights advantages of using single-cell pHi measurements to investigate single-cell behaviors like cell cycle progression, and suggests mechanisms to limit pHi-dependent cell cycle progression in diseases with dysregulated pH, such as cancer (increased pHi) (Harguindey et al., 2017; White et al., 2017a) and neurodegeneration (decreased pHi) (Majdi et al., 2016).

## RESULTS

### Single-cell pHi in clonal cell lines is heterogeneous

**Fig. 1.**
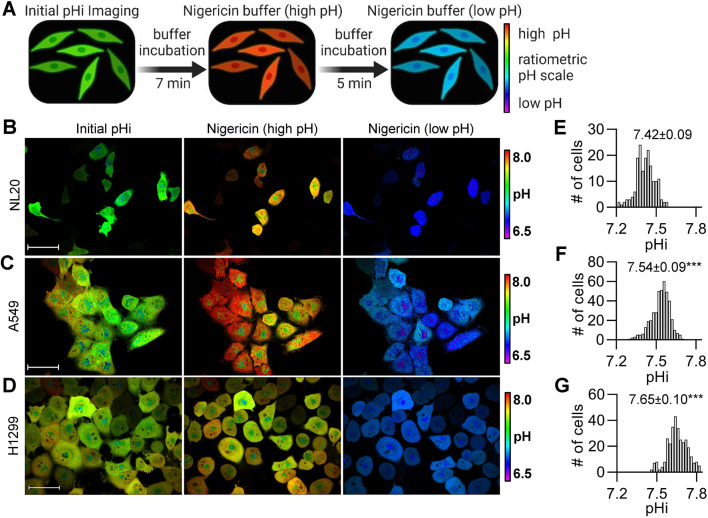
**Intracellular pH is heterogeneous in normal and cancerous lung cell lines and median pHi significantly increases in cancer cells.** (A) Schematic of single-cell pHi measurements using a stably expressed pH biosensor, mCherry–pHluorin (mCh-pHl), and the protonophore nigericin to standardize the biosensor (see Materials and Methods for details). (B–D) Representative images of pHi measurements and standardization in (B) NL20, (C) A549, and (D) H1299 cells stably expressing mCh-pHl. Ratiometric display of pHluorin/mCherry fluorescence ratios. Scale bars: 50 μm. (E–G) Histograms of single-cell pHi in (E) NL20 (*n*=173, three biological replicates), (F) A549 (*n*=424, four biological replicates) and (G) H1299 (*n*=315, three biological replicates). Histograms are binned at 0.02 pH units. Above histograms, median±interquartile range is shown. Significance was determined by a Mann–Whitney test (***P<0.001 compared to NL20).

We first examined whether asynchronous single-cell pHi measurements under physiological conditions could recapitulate population-level averages, while also reporting on physiological single-cell heterogeneity. We used a genetically-encoded pH biosensor, mCherry–pHluorin (mCh-pHl) (Koivusalo et al., 2010), which has been used to measure pHi in cultured cells (Choi et al., 2013; Koivusalo et al., 2010) and tissues (Grillo-Hill et al., 2015) and has a dynamic linear range between pH 6.5 and 8.0 (Grillo-Hill et al., 2014). Briefly, direct measurement of pHi in single living cells can be achieved by performing ratiometric imaging of pHluorin and mCherry fluorescence intensities. Fluorescence of pHluorin is pH-sensitive in the physiological range, whereas mCherry fluorescence is pH-insensitive and used to normalize for biosensor expression. At the end of the experiment, single-cell standardization is performed using isotonic buffers of known pH containing the protonophore nigericin (Fig. 1A). This method of pHi measurement avoids issues of uneven dye loading, washout and photobleaching associated with pH-sensitive dyes (Grillo-Hill et al., 2014).

We stably expressed mCh-pHl in normal lung epithelial cells (NL20), primary tumor site-derived lung cancer cells (A549) and metastatic site-derived lung cancer cells (H1299). We chose lung-derived cells because these clonal cell lines are well characterized in the literature, are morphologically heterogeneous, and tolerate stable expression of the mCh-pHl biosensor. We first confirmed that biosensor expression does not alter pHi homeostasis in these cells by comparing population pHi measurements of the clonal biosensor lines (NL20-mCh-pHl, A549-mCh-pHl and H1299-mCh-pHl) to those of matched parental cell lines (Grillo-Hill et al., 2014) (Fig. S1A–C). One distinct advantage of single-cell imaging experiments for pHi measurement is that pHi can be measured directly in conditioned medium without the need to use fresh bicarbonate- or HEPES-based isotonic washing solutions that are required for population level assays (see Materials and Methods for solution composition). Thus, single-cell pHi measurements are more likely to reflect accurate pHi setpoints and dynamics of cells and give better comparison to other cell biological assays or signaling profiles measured from cells cultured continuously in complete medium.

We next measured single-cell pHi in individual NL20-mCh-pHl (Fig. 1B), A549-mCh-pHl (Fig. 1C) and H1299-mCh-pHl (Fig. 1D) cells. Representative pHluorin and mCherry fluorescence images and single-cell standardization curves can be found in Fig. S1D–I. To assay pHi heterogeneity in these clonal cell lines, we prepared distribution histograms of single-cell pHi measurements and found that the pHi of primary tumor cells (A549-mCh-pHl) (Fig. 1F; 7.54±0.09; median±interquartile range) was increased compared to normal lung epithelial cells (NL20-mCh-pHl) (Fig. 1E; 7.42±0.09). Importantly, metastatic tumor cells (H1299-mCh-pHl) had the highest median pHi (Fig. 1G; 7.65±0.10), which was significantly higher than both the normal and primary tumor clonal cell lines. To support these results, we also measured single-cell pHi in metastatic triple-negative breast cancer cells (MDA-MB-231) and found it was also significantly increased (7.52±0.19) compared to pHi in matched normal breast epithelial cells (MCF10A) (7.23±0.26) (Fig. S2). Taken together, we find that aggressive cancer cell lines have higher single-cell pHi compared to normal epithelial cells across multiple tissue origins. Importantly, our data show that pooled single-cell pHi measurements reveal significant heterogeneous pHi distributions that are lost in population-level analyses. These data also suggest that even genetically identical clonal cell lines exhibit single-cell pHi heterogeneity that might be biologically meaningful and could report on non-genetic cell phenotype such as cell cycle status.

### Cells released from G1 synchronization have dynamic pHi

**Fig. 2.**
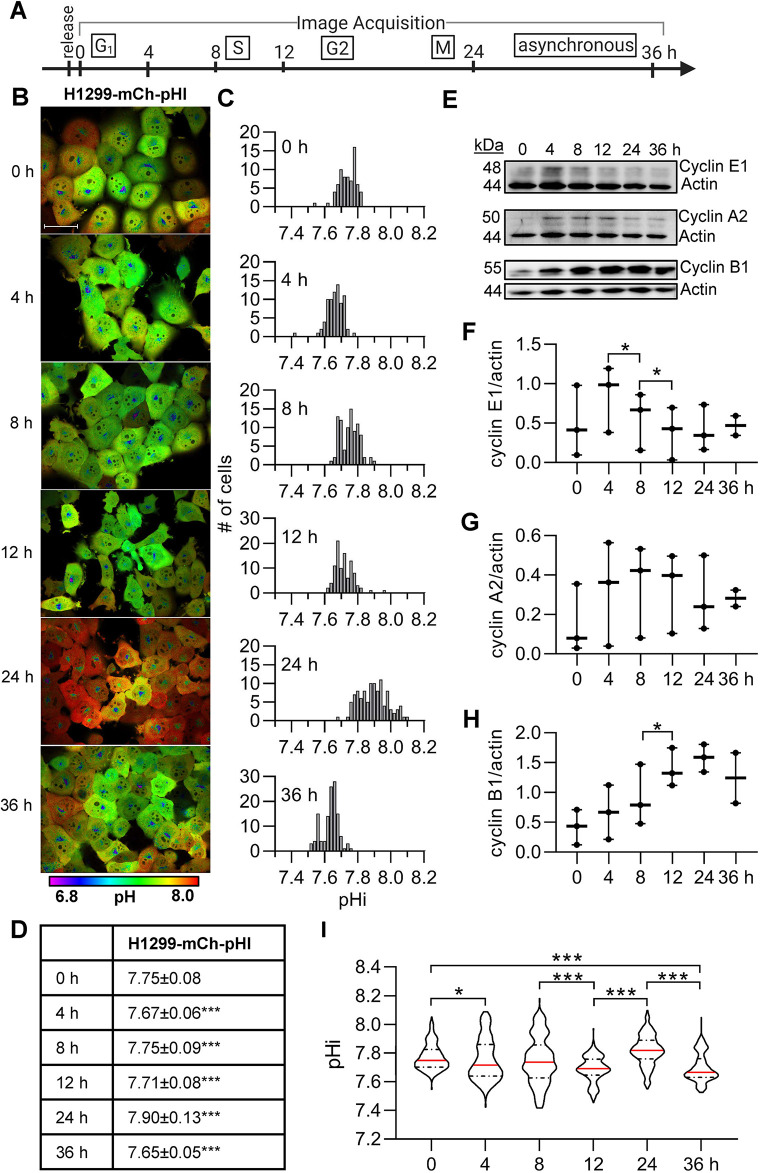
**Intracellular pH is dynamic following G1 synchronization and correlates with cyclin levels.** (A) Schematic of image acquisition after palbociclib synchronization. (B) Representative images of H1299-mCh-pHl cells at indicated time points after release. Ratiometric display of pHluorin/mCherry fluorescence ratios. Scale bar: 50 μm. (C) Histograms of single-cell pHi data collected as in B, from one biological replicate. Histograms binned at 0.02 pH units. Additional replicates in Fig. S3. (D) Table of pHi values from data in C (median±interquartile range). (E) Representative immunoblots for cyclin E1, A2, and B1 with actin loading controls. Scatter plots of (F) cyclin E1, (G) cyclin A2, and (H) cyclin B1 immunoblot data (three biological replicates; median and range indicated). Additional replicates in Fig. S3. (I) Violin plots of raw pHi (0 h, *n*=231; 4 h, *n*= 253; 8 h, *n*=262; 12 h, *n*=273; 24 h, *n*=338; 36 h, *n*=262; three biological replicates). Red lines are the median and dashed lines mark quartiles. In D and I, significance was determined by Kruskal–Wallis test with Dunn's multiple comparisons correction. In F–H, significance was determined by paired two-tailed *t*-test. In D and F–I, each time point was compared to the preceding time point and, in I, 0 h was additionally compared to 24 h (*P<0.05; ***P<0.001).

Next, we sought to measure pHi dynamics during cell cycle progression. We synchronized H1299-mCh-pHl cells using palbociclib, which blocks phosphorylation of the retinoblastoma protein and synchronizes cells prior to the G1 checkpoint (Liu et al., 2018) (Fig. 2A). Palbociclib is an efficient G1 synchronizer in H1299 cells, with nearly 85% synchronization after 24 h treatment and minimal DNA damage (Trotter and Hagan, 2020). After palbociclib synchronization, cells were imaged at 0, 4, 8, 12, 24 and 36 h after release (Fig. 2B) and single-cell pHi distributions were measured (Fig. 2C). Qualitatively, we noticed that cells were larger at earlier time points (0–4 h) and that, at 12 h, cells had cell rounding and smaller apparent cell size indicative of mitotic cells (Fig. 2B). We observed oscillating pHi distributions during cell cycle progression where single-cell pHi significantly decreased between 0 and 4 h, significantly increased between 4 and 8 h, decreased again between 8 and 12 h, and finally increased between 12 and 24 h (Fig. 2D). These data suggest that pHi is dynamic during cell cycle progression with temporally regulated fluctuations in pHi after synchronization release.

We confirmed that palbociclib appropriately synchronized the cells by immunoblotting for cyclins from matched cell lysates (Fig. 2E–H). Cyclin E1 regulates G1/S (Siu et al., 2012), cyclin A2 regulates S and G2 phases (De Boer et al., 2008), and cyclin B1 regulates G2 and must be degraded prior to anaphase in mitosis (Chang et al., 2003). We observed that cyclin E1 levels, a marker of G1/S, significantly increased from 0 to 4 h, which is expected for a cell population properly synchronized in G1 phase (Fig. 2F). These cells undergo mitosis ∼24 h post-palbociclib release because cyclin A2 levels, a marker of S/G2, were dropping at 24 h (Fig. 2G) and cyclin B1 levels, an inducer of G2/M, peaked 24 h post-release (Fig. 2H). By 36 h, protein abundance was similar across all cyclins, as expected in an asynchronous population. Cyclin immunoblots and pHi agreed across three biological replicates (additional pHi replicates and blots in Fig. S3).

Single-cell pHi measurements on palbociclib-treated cells were compared over three biological replicates, and we found that pHi significantly decreased at the G1/S transition (4 h, 7.72±0.22; median±interquartile range) and in late S phase (12 h, 7.69±0.11), significantly increased at G2/M (24 h, 7.82±0.13), and then significantly decreased again at the end of the experiment in asynchronous cells (36 h, 7.67±0.13) (Fig. 2I). To assess whether palbociclib treatment alters resting pHi, we pooled synchronized cells from all the different time points (Fig. 2I, 0–24 h) and compared data from these pooled cells to pHi measurements at experiment endpoint (Fig. 2I, 36 h) and to untreated asynchronous H1299-mCh-pHl pHi data (Fig. 1G). We note that pHi in palbociclib-treated cells was significantly increased compared to untreated asynchronous cells, indicating that palbociclib synchronization might also alter pHi homeostasis (Fig. S3C). Previous work did find that palbociclib induced markers of senescence and autophagy when used for >36 h (Capparelli et al., 2012), so this is a confounding factor on pHi at 24 h of treatment. However, the increases in resting pHi were uniform in our data and trends in pHi dynamics were robust across multiple biological replicates.

### Cells exhibit cell cycle-linked pHi dynamics independently of the cell cycle synchronization method

**Fig. 3.**
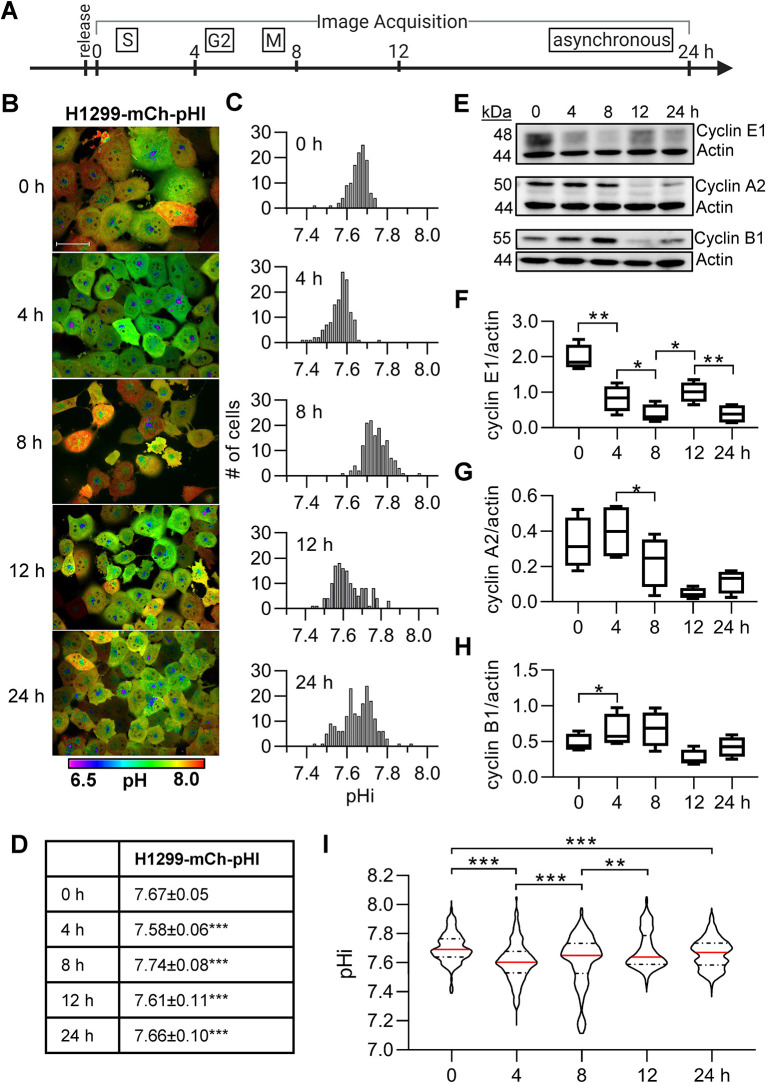
**Intracellular pH is dynamic after release from early S phase in H1299-mCh-pHl cells and correlates with cyclin levels.** (A) Schematic of image acquisition after a double-thymidine synchronization. (B) Representative images of H1299-mCh-pHl cells at indicated time points after release. Ratiometric display of pHluorin/mCherry fluorescence ratios. Scale bar: 50 μm. (C) Histograms of single-cell pHi data collected in B, from one biological replicate. Histograms binned at 0.02 pH units. Additional replicates in Fig. S4. (D) Table of pHi values from data in C (median±interquartile range). (E) Representative immunoblots for cyclin E1, A2, and B1 with respective actin loading controls. Box-and-whisker plots of (F) cyclin E1, (G) cyclin A2, and (H) cyclin B1 immunoblot data (4 biological replicates). Additional replicates in Fig. S4. Median indicated by line, the box shows the 25–75th percentiles, and the whiskers show minimum and maximum values. (I) Violin plots of raw pHi values (0 h, *n*=500; 4 h, *n*= 468; 8 h, *n*=517; 12 h, *n*=558; 24 h, *n*=652; 4 biological replicates). Red lines are the median and dashed lines mark quartiles. In D and I, significance was determined by a Kruskal–Wallis test with Dunn's multiple comparisons correction. In F–H, significance was determined by a paired two-tailed *t*-test. In D and F–I, each time point was compared to its preceding time point and in I, 0 h was additionally compared to 24 h (*P<0.05; **P<0.01; ***P<0.001).

To confirm that the temporal pHi dynamics observed in Fig. 2 were linked to specific cell cycle phases and were not an artifact, or off-target effect, of palbociclib synchronization, we next synchronized H1299-mCh-pHl cells in early S phase using a double-thymidine block (Chen and Deng, 2018). Thymidine acts as a DNA synthesis inhibitor by accumulating dTTP and depleting dCTP within the cell (Bjursell and Reichard, 1973; Bolderson et al., 2004). We synchronized H1299-mCh-pHl cells and imaged them at 0, 4, 8, 12 and 24 h after thymidine release (Fig. 3A,B). Qualitatively, cells were larger at 0 h and, by 8 h, had altered morphology that could indicate mitosis (Fig. 3B). In this representative replicate, single-cell pHi significantly decreased between 0 and 4 h, significantly increased between 4 and 8 h, decreased between 8 and 12 h, and increased again between 12 and 24 h (Fig. 3C,D). This general trend supports pHi data from cells released earlier in the cell cycle (palbociclib, G1) (Fig. 2). Importantly, the observed phase-shifted pHi oscillations confirm that pHi dynamics are linked to cell cycle timing and not experiment timing, regardless of the synchronization method used.

We also confirmed that thymidine treatment appropriately synchronized the cells by immunoblotting for cyclins from matched cell lysates (Fig. 3E). We found that cyclin E1 (G1/S) peaks at 0 h, as expected for a cell population synchronized in early S phase (Fig. 3F). The cells undergo mitosis ∼8 h after thymidine release as cyclin A2 (S/G2) was highest at 4 h and significantly decreased by 8 h (Fig. 3G), whereas cyclin B1 (G2/M) peaked 8 h after release (Fig. 3H). Cyclin E1 levels increased again by 12 h, suggesting that by 12 h most cells in this assay had completed the cell cycle and progressed back to G1 (Fig. 3F). By 24 h, protein abundance was similar across all cyclins, as we would expect in an asynchronous population. Immunoblots for additional replicates are shown in Fig. S4, and pooled cyclin results match previously published data on synchronized H1299 cell populations (Chen and Deng, 2018).

Single-cell pHi measurements from pooled thymidine-treated biological replicates revealed that the median pHi of the cell populations decreased significantly at 4 h (late S phase), increased from 4 to 8 h (G2/M), and decreased again from 8 to 12 h (M/G1) (Fig. 3I). This matches the oscillating pHi pattern measured in the individual replicate (Fig. 3B,C; additional replicates in Fig. S4). Thymidine synchronization did not alter homeostatic pHi when compared to pooled synchronized thymidine data (Fig. 3I, 0–12 h), asynchronous thymidine data (Fig. 3I, 24 h) or untreated H1299-mCh-pHl cells (Fig. 3G) (see also Fig. S4C). The single-cell pHi data after thymidine release confirms both the decreased pHi in late S phase and increased pHi at G2/M that we measured after palbociclib release, and also reveals a significant pHi increase during early S phase.

To confirm that cell cycle-linked pHi dynamics are not unique to H1299 cells, we synchronized A549-mCh-pHl cells with a double-thymidine block and observed similar pHi dynamics (Fig. S5). Cell morphology and pHi oscillations matched those of H1299-mCh-pHl cells at respective time points (Fig. S5A), with a decrease in pHi from 0 h to 4 h, an increase from 4 h to 8 h, and decreases at 12 h and 24 h (Fig. S5B,C). Again, synchronization was confirmed using cyclin immunoblots, where cyclin A2 (S/G2) peaked at 4 h, cyclin B1 (G2/M) peaked at 8 h, and both proteins were low at 12 h indicating the start of a new cycle with cells in G1 (Fig. S5D). Like H1299-mCh-pHl cells, the pHi of pooled A549-mCh-pHl cells significantly increased from 4 h to 8 h (G2/M) and decreased following mitosis at 12 h (Fig. S5E). Observing identical cell cycle-linked pHi dynamics across different cell models suggests pHi increases prior to division (G2/M, 4–8 h) and decreases after division (G1, 8–12 h) might be necessary for division timing and re-entry into the cell cycle.

From these data, we conclude that pHi is dynamic through the cell cycle at the single-cell level: pHi decreases during G1/S, increases in early S phase, decreases leading to S/G2, increases prior to G2/M and decreases following mitosis.

### Single cells alkalize prior to G2/M and rapidly acidify during mitosis, followed by pHi recovery in daughter cells

In the previous experiments, cells from matched populations were identically treated and released from synchronization for imaging at various time points after release. These time points showed that single-cell pHi distributions oscillate with cell cycle progression, but the snapshots might not reflect continuous single-cell pHi dynamics or cell cycle progression phenotypes. To address this limitation, we established a time-lapse microscopy approach to track pHi dynamics over an entire cell cycle in a single cell.

**Fig. 4.**
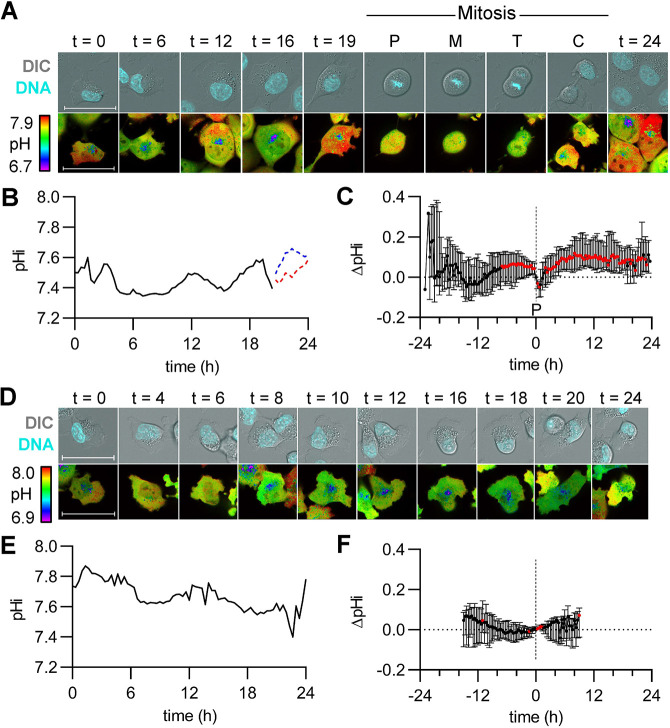
**Intracellular pH increases leading to G2/M, followed by rapid acidification prior to division and pHi recovery in daughter cells.** (A) Representative stills from Movie 1 of a dividing H1299-mCh-pHl cell at indicated time (h). Top is Hoechst 33342 dye (DNA, cyan) and DIC merge. Bottom is ratiometric display of pHluorin/mCherry fluorescence ratios. Scale bars: 50 μm. Labels indicate prophase (P), metaphase (M), telophase (T), and cytokinesis (C). (B) Traces of calculated pHi values of the parent cell in A (black, solid line) and in daughter cells (red and blue dotted lines). (C) pHi changes in dividing cells, relative to pHi at prophase (P, vertical dashed line) for each individual cell (median±interquartile range, *n*=39, four biological replicates). Significance was determined by a one-sample Wilcoxon test compared to 0 (red points are *P*<0.05). (D) Representative stills from Movie 2 of a non-dividing H1299-mCh-pHl cell at indicated time (h). Top is Hoechst 33342 dye (DNA, cyan) and DIC merge. Bottom is ratiometric display of pHluorin/mCherry fluorescence ratios. Scale bars: 50 μm. (E) Trace of pHi values of cell in D (black, solid line) over time. (F) pHi changes in non-dividing cells, relative to pHi for each individual cell at experimental time t=15 h (vertical dashed line) (median±interquartile range, *n*=25, four biological replicates). Significance was determined by a one-sample Wilcoxon test compared to 0 (red points are *P*<0.05).

We first measured pHi changes in single asynchronous H1299-mCh-pHl cells. We observed cells randomly dividing throughout the time-lapse, indicating the cells were asynchronous (Fig. S6A), and representative stills of ratiometric time-lapse pHi imaging in a dividing cell are shown (Fig. 4A; Movie 1). For this cell, pHi quantification shows oscillating pHi dynamics similar to those observed in the snapshot experiments (Fig. 4B). Although we were able to mark entry into mitosis via DNA condensation at prophase, we cannot determine other cell phase transitions with this approach. We noted there is a prominent alkalization in the hours prior to mitosis (Fig. 4A,B, 19 h), followed by rapid acidification during mitosis (Fig. 4A,B, labels P, M, T and C). To compare trends in single-cell pHi dynamics across many individual cells, we selected prophase as a ‘normalization point’ for each individual dividing cell. We observed a significant period of alkalization that began ∼7 h prior to division and persisted until prophase (Fig. 4C). These pHi increases in single dividing cells correlate with the increased pHi observed during G2/M in the discontinuous endpoint data (Figs 2I and 3I; Fig. S5E) and suggest that increased pHi might be a required signal for division of single cells. The single-cell time-lapse analysis also allows us to distinguish cells that undergo mitosis and cells that do not. If pHi dynamics are a sufficient regulator of cell cycle progression, we might expect to see attenuated pHi dynamics in non-dividing cells. To test this hypothesis, we quantified pHi in non-dividing cells. Representative stills of ratiometric time-lapse pHi imaging in a non-dividing cell from the asynchronous population are shown (Fig. 4D; Movie 2). We found that pHi dynamics are attenuated in non-dividing cells compared to dividing cells (Fig. 4E,F), suggesting that pHi dynamics are correlated with successful cell cycle progression. Thus, pHi dynamics might be an important biomarker for or driver of normal cell cycle progression.

**Fig. 5.**
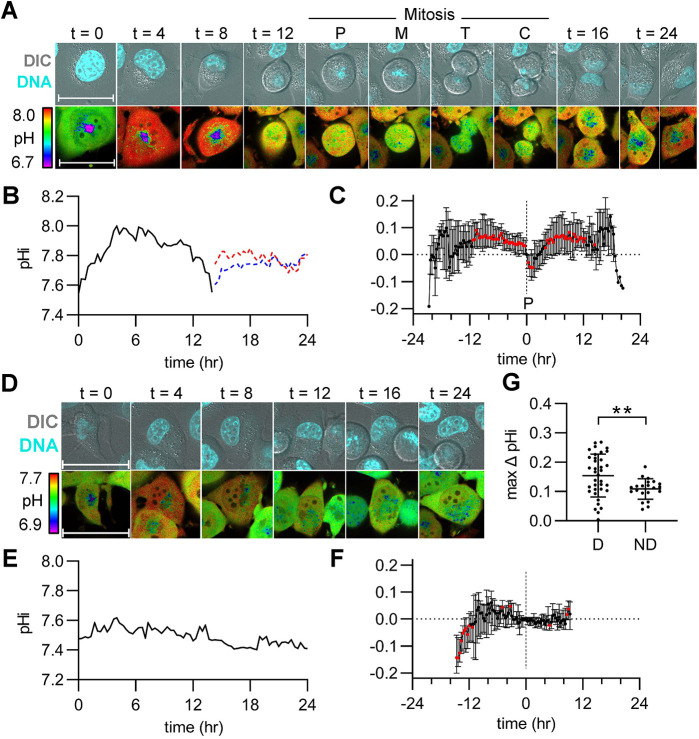
**Cells released from S phase synchronization show pHi increases, leading to G2/M, rapid acidification prior to division and pHi recovery of daughter cells.** (A) Representative stills from Movie 3 of a dividing H1299-mCh-pHl cell at indicated time (h). Top is Hoechst 33342 dye (DNA, cyan) and DIC merge. Bottom is ratiometric display of pHluorin/mCherry fluorescence ratios. Scale bars: 50 μm. Labels indicate prophase (P), metaphase (M), telophase (T), and cytokinesis (C). (B) Traces of calculated pHi values of the cell in A (black, solid line) and in daughter cells (red and blue dotted lines). (C) pHi changes in dividing cells, relative to pHi at prophase (P, vertical dashed line) for each individual cell (median±interquartile range, *n*=39, three biological replicates). Significance was determined by a one-sample Wilcoxon test compared to 0 (red points are *P*<0.05). (D) Representative stills from Movie 4 of a non-dividing H1299-mCh-pHl cell at indicated time (h). Top is Hoechst 33342 dye (DNA, cyan) and DIC merge. Bottom is a ratiometric display of pHluorin/mCherry fluorescence ratios. Scale bars: 50 μm. (E) Trace of pHi values of cell in D (black, solid line) over time. (F) pHi changes in non-dividing cells, relative to pHi for each individual cell at experimental time t=15h (vertical dashed line), the average time of prophase for dividing cells in this dataset (median±interquartile range, *n*=22, 3 biological replicates). Significance was determined by a one-sample Wilcoxon test compared to 0 (red points are *P*<0.05). (G) Scatter plot of max pHi change in individual dividing (D) and non-dividing (ND) cells (mean±s.d.). Significance was determined by an unpaired two-tailed *t*-test (**P<0.01).

In order to directly compare single-cell time-lapse pHi dynamics to the prior data, we next collected time-lapse pHi measurements in thymidine-synchronized H1299-mCh-pHl cells. We observed bursts of mitotic cells at 15 h, indicating that thymidine was appropriately synchronizing individual cells (Fig. S6B), and representative stills of ratiometric time-lapse pHi imaging in a dividing cell are shown (Fig. 5A; Movie 3). For this dividing cell, pHi increased through late S and G2 phases (matched to Fig. 3 cyclin timing data), decreased during mitosis and recovered rapidly in daughter cells (Fig. 5B). Similar to what was undertaken in the asynchronous time-lapse data above, we used prophase as a ‘normalization point’ for comparing pHi in individual dividing cells, and observed oscillating pHi dynamics with a significant period of alkalization beginning ∼11 h prior to division and persisting until prophase, followed by a rapid acidification during mitosis and recovery in daughter cells (Fig. 5C). Non-dividing cells had significantly attenuated pHi dynamics compared to dividing cells at the single-cell level (Fig. 5D,E; Movie 4). To compare trends in single-cell pHi dynamics for non-dividing cells, the change in pHi was calculated for each non-dividing cell from the average prophase time in the synchronized time-lapses (15 h). We again observed attenuated pHi dynamics in single non-dividing cells (Fig. 5F). Importantly, the extended alkalization observed prior to prophase (Figs 4C and 5C) was not observed in either non-divider dataset. This suggests that dynamic pHi could be a hallmark for cells moving through the cell cycle.

Given that cell synchronization confers increased consistency of cell phase transitions, we assigned the thymidine data to bins depending on timing of prophase (Fig. S6B). Importantly, we observed significant alkalizations in each group ∼5 h prior to prophase regardless of mitosis timing (Fig. S6C), which correlates with G2 entry based on previous data in H1299 cells (Rajal et al., 2021) and our own data with FUCCI cell cycle reporters (shown below; G2 is ∼4.0 h). These data strongly suggest that the increased pHi we observe across all time-lapse datasets at 5 h coincides with G2 entry. Next, to confirm that the dynamics we observed in the time-lapse are not an artifact of increased mCh-pHl biosensor expression or altered biosensor photobleaching rates, we tracked mCherry and pHluorin intensities over time in both dividing and non-dividing cells from the synchronous time-lapses (Fig. S6D,E). We observed that mCherry fluorescence dynamics in dividing cells showed similar trends compared to non-dividing cells across the time-lapse experiment (Fig. S6D). Furthermore, the pHluorin increases observed over time in dividing cells were not correlated with increased mCherry fluorescence, indicating observed pHluorin increases are not due to increases in biosensor expression (Fig. S6E) but instead reflect dynamic pHi in single cells.

These time-lapse data suggest that increased single-cell pHi dynamics might be correlated with (or regulate) single-cell cell cycle progression. Supporting this hypothesis, parent cells that divided within the 24 h period showed a significantly higher mean pHi increase (0.15±0.07, mean±s.d.; Fig. 5G) when compared to that of non-dividing cells (0.11±0.03; Fig. 5G). We note that the magnitude of pHi changes observed in single dividing cells (∼0.15 pH units) corresponds well with physiological pHi increases previously reported in single cells during other cell behaviors, such as cell migration (0.1–0.35) (Denker and Barber, 2002).

Taken together, these time-lapse data suggest that single dividing cells have oscillating pHi dynamics with an increase in pHi in the time leading up to mitosis, a rapid acidification during mitosis and recovered pHi in daughter cells. Our next question was whether pHi dynamics regulate or time cell cycle progression.

### Dysregulated pHi dynamics affect cell cycle phase duration and cause phase-specific arrests

**Fig. 6.**
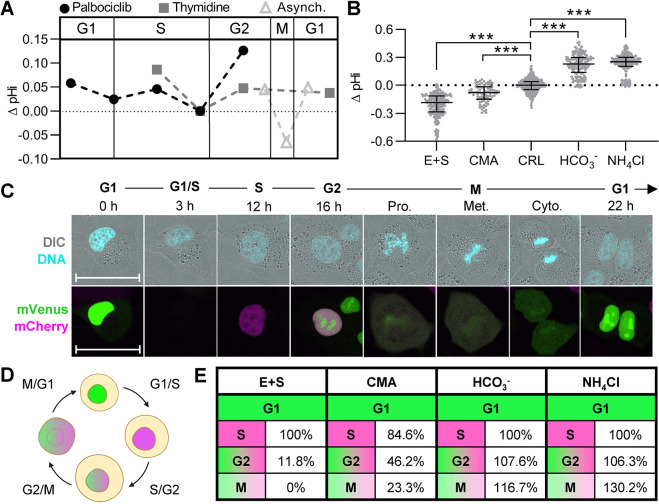
**Single-cell pHi manipulation shows that pHi dynamics are key regulators of the cell cycle.** (A) Median plots of single-cell ΔpHi from synchronizations and asynchronous (Asynch.) time-lapses. Data reproduced from Figs 2I, 3I, and 4C (thymidine, normalized to 4 h, *n*=4; palbociclib, normalized to 12 h, *n*=3; Asynch., normalized to prophase, *n*=4). (B) Single-cell pHi of H1299-mCh-pHl cells treated for 24 h with 15 μM EIPA and 30 μM S0859 (E+S, *n*=233) or 1 μM concanamycin A (CMA, *n*=79) to lower pHi, untreated (CRL, *n*=602), or supplemented with 100 mM NaHCO_3_ (HCO_3_^−^, *n*=146) or 20 mM ammonium chloride (NH_4_Cl, *n*=193) to raise pHi (see Materials and Methods for details). Additional treatment time points are shown in Fig. S8. (C) Representative stills from Movie 5. Shown is a single H1299-FUCCI cell with PIP–mVenus (green) and mCherry–Geminin (magenta) tracked through each cell cycle phase. Hoechst 33342 dye (DNA, cyan) and DIC merge shown. Scale bars: 50 μm. (D) Schematic of PIP-FUCCI reporter fluorescence during cell cycle phase transitions (Grant et al., 2018). (E) Successful phase entry of cells starting in G1, where each treatment is normalized to matched controls. E+S (*n*=27), CMA (*n*=13), HCO_3_^−^ (*n*=13), NH_4_Cl (*n*=13). For B, scatter plots (median±interquartile range), with Mann–Whitney test to determine statistical significance (***P<0.001).

We have shown pHi is dynamic and correlates with cell cycle phases in asynchronous cells, cells synchronized at G1 with palbociclib (Fig. 2) and cells synchronized at early S phase with thymidine (Fig. 3). To compare pHi data from both synchronization techniques, we aligned pHi data (Figs 2I and 3I) according to significantly increased cyclin B1 expression (Figs 2H and 3H) and found oscillating pHi dynamics throughout the cell cycle (Fig. 5A). We observed that pHi decreases during G1/S, increases in mid-S phase, decreases prior to S/G2 and increases prior to G2/M (Fig. 6A). In addition, the time-lapse data in asynchronous (Fig. 4C) and thymidine-synchronized single cells (Fig. 5C) confirmed the dynamic increases in pHi leading to G2/M and uncovered a rapid acidification during M phase (Fig. 6A). These data suggest a correlation between pHi and cell cycle progression, but to determine a causal relationship, we sought to manipulate pHi and monitor effects on cell cycle phases in real-time. To do this, we established pHi manipulation techniques (Larsen et al., 2012; White et al., 2017b) and used the FUCCI cell cycle reporter (Grant et al., 2018) to track single cells during cell cycle progression.

To manipulate pHi, we used combinations of selective ion transporter inhibitors to lower pHi and medium supplementation to raise pHi. To lower pHi, we used concanamycin A (CMA), which inhibits V-ATPases (Huss et al., 2002), 5-(N-ethyl-N-isopropyl)amiloride (EIPA), which inhibits NHE1 (White et al., 2017b), and 2-chloro-N-[[2′-[(cyanoamino)sulfonyl][1,1′-biphenyl]-4-yl]methyl]-N-[(4-methylphenyl)methyl]-benzamide (S0859) (Larsen et al., 2012), which inhibits the Na^+^-HCO_3_^−^ transporter (NBCn1) (see Materials and Methods for details). Both incubation with CMA and combination treatment with EIPA and S0859 (E+S) lowered pHi compared to CRL (lowering pHi by ∼0.08 and ∼0.18 pH units, respectively; Fig. 6B). To raise pHi, we supplemented the medium with ammonium chloride (NH_4_Cl) or with bicarbonate (HCO_3_^−^) (raising pHi by ∼0.25 and ∼0.23 pH units compared to CRL, respectively; Fig. 6B).

FUCCI reporters use regulatory domains of cell cycle proteins to differentially express fluorescent proteins and report on cell cycle progression in single cells (Fig. 6C,D). We used the PIP-FUCCI reporter, which allows improved delineation of S phase (Grant et al., 2018) compared to older FUCCI variants. PIP-FUCCI reporter fluorescence is driven by the regulatory domains of the PCNA-interacting protein degron from human Cdt1 (amino acids 1–17; denoted PIP) fused to mVenus, and Geminin_1-110_ fused to mCherry. PIP–mVenus accumulates in the nucleus during G1 and is rapidly lost during the onset of DNA replication (S phase). At the beginning of S phase, mCherry–Geminin accumulates and is expressed throughout S, G2, and M phases. During the S/G2 transition, mVenus accumulates again, and both mVenus and mCherry are co-expressed until division. Thus, the PIP-FUCCI reporter system enables accurate delineation of both G1/S and S/G2. M phase is marked by nuclear envelope breakdown and diffusion of mVenus and mCherry fluorescent proteins throughout the cell. Mitosis and cytokinesis can also be monitored through DNA staining and DIC imaging (Fig. 6C). Following cytokinesis, only mVenus is expressed in the two daughter cell nuclei, marking G1.

To determine how pHi dynamics regulate cell cycle progression, we stably expressed PIP-FUCCI in H1299 cells (H1299-FUCCI) (Fig. 6C; Movie 5) and applied the validated pHi manipulation techniques to experimentally raise and lower pHi in cells (Fig. 6B). Using time-lapse confocal microscopy, we tracked single cells (with and without pHi manipulation) over a 36 h period and analyzed mVenus and mCherry fluorescent intensities to determine successful progression of single-cell cell cycle phases (see Materials and Methods for details).

First, we analyzed the successful transition of cells from G1 to subsequent cell cycle phases in each pHi manipulation treatment compared to untreated (CRL) cells. We observed some common effects of pHi manipulation on the ability of single cells to progress normally through the cell cycle. First, successful S/G2 transitions were reduced when pHi was lowered with either EIPA E+S (11.8%) or CMA (46.2%) and increased when pHi was raised with either HCO_3_^−^ (107.6%) or NH_4_Cl (106.3%) (all compared to CRL, Fig. 6E). Second, successful G2/M transitions were similarly reduced when pHi was lowered with E+S (0%) or CMA (23.3%) and increased when pHi was raised with either HCO_3_^−^ (116.7%) or NH_4_Cl (130.2%) (all compared to CRL, Fig. 6E). We also note that the phenotype of successful phase transitions correlated with the magnitude of pHi changes with low pHi manipulation – larger decreases in pHi induced by E+S produced stronger phenotypes compared to smaller pHi decreases induced by CMA. These data indicate that pH dynamics do regulate successful cell cycle phase transitions, where decreased pHi is detrimental to S/G2 and G2/M transitions and increased pHi promotes these transitions in single cells.

We next wanted to explore how pHi manipulation alters the length of cell cycle phases. We first tested whether the selected pHi manipulation approaches induce replicative stress, as it has been previously shown that replication stress can cause cell cycle phase dysregulation (Matthews et al., 2022; Técher et al., 2017). We treated cells with each of the pHi manipulation techniques (Fig. 6B) and stained the cells for phosphorylated H2AX (γ-H2AX), a common marker of replicative stress (Kuo and Yang, 2008). We found that CMA alone significantly increased γ-H2AX staining, so we removed that treatment condition from further analysis (Fig. S7). Although prior work has shown that high concentrations of EIPA can induce γ-H2AX (Rolver et al., 2020), we did not observe induction of γ-H2AX with E+S in our system (Fig. S7). We next considered whether altered metabolism contributed significantly to the observed results. Although supplementation with HCO_3_^−^ raised pHi (Fig. 6B), it also alters extracellular pH (which we noted by observing a change in the Phenol Red indicator in the HCO_3_^−^-treated medium) and drastically alters cellular metabolism (Krycer et al., 2017; Li et al., 2020), which can dysregulate cell cycle phases independently of pHi (Mitaka et al., 1991). For this reason, we also did not continue with full quantification of phase length with HCO_3_^−^ treatment.

**Fig. 7.**
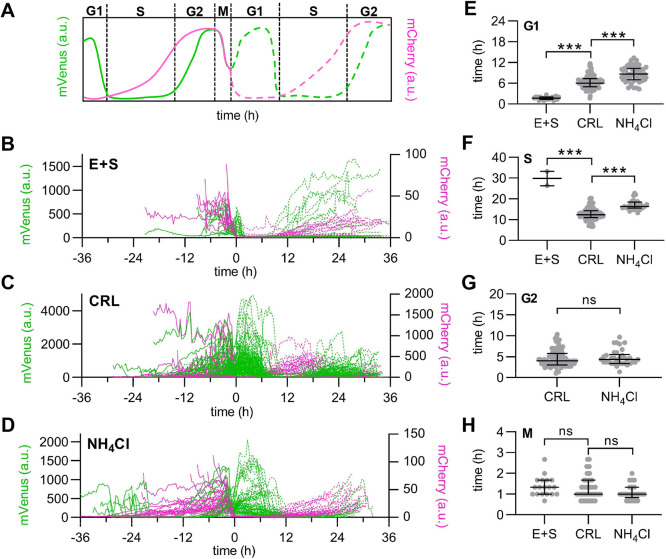
**Single-cell FUCCI traces show low pHi is a cue for G1 exit, S phase requires high and low pHi, and S/G2 requires high pHi.** (A) Schematic of PIP–mVenus (green) and mCherry–Geminin (magenta) fluorescence intensities during cell cycle phases. (B–D) Traces from single H1299-FUCCI cells treated as in Fig. 6B (E+S, 15 μM EIPA plus 30 μM S0859; NH_4_Cl, 20 mM NH_4_Cl). Traces aligned at time of division at 0 h, and daughter cells are indicated by dotted lines: (B) E+S, (*n*=23); (C) CRL (*n*=187); (D) NH_4_Cl (*n*=72) (CRL and NH_4_Cl, three biological replicates; E+S, two biological replicates). In A–D: a.u., arbitrary units. (E–H) Cell cycle phase durations from all cell populations (dividers and non-dividers). (E) G1 (E+S, *n*=22; CRL, *n*=151; NH_4_Cl, *n*=51), (F) S (E+S, *n*=3; CRL, *n*=88; NH_4_Cl, *n*=26), (G) G2 (CRL, *n*=90; NH_4_Cl, *n*=34), and (H) M (E+S, *n*=18; CRL, *n*=113; NH_4_Cl, *n*=33). For E–H, scatter plots (median±interquartile range), with Mann–Whitney test to determine statistical significance (***P<0.001; ns, not significant).

One benefit of using FUCCI reporters is that they can be used to assay cell cycle phase completion but can also be used to directly measure cell cycle phase length (Fig. 7A). To quantify cell cycle phase length, we plotted single-cell traces of FUCCI fluorescence intensities (mVenus and mCherry) aligned to division time (Fig. 7B–D). Importantly, we validated that even within 4 h of treatment the pHi manipulations were sufficient to significantly change pHi (Fig. S8). Using fluorescence intensity cutoffs to determine G1/S and S/G2 (Fig. 7A, see Materials and Methods), we measured significant differences in phase durations with high and low pHi conditions (Fig. 7E–H). From the single-cell traces, we first noted that G1 phase in daughter cells (dotted lines, after 0 h) was altered in both E+S- and NH_4_Cl-treated cells compared to control. G1 phase (high mVenus and low mCherry) was significantly shortened in daughter cells at low pHi (Fig. 7E, 1.7±0.7 h, median± interquartile range) and significantly elongated at high pHi (Fig. 7E, 8.7±3.3 h) compared to that in CRL cells (Fig. 7E, 6.0±2.3 h). These pHi-dependent changes in G1 phase duration indicate that low pHi might be a cue for G1 exit and that aberrant alkalization delays this cell cycle transition. These data align with the measured decreased pHi at G1/S in the prior endpoint assays (Fig. 6A).

**Fig. 8.**
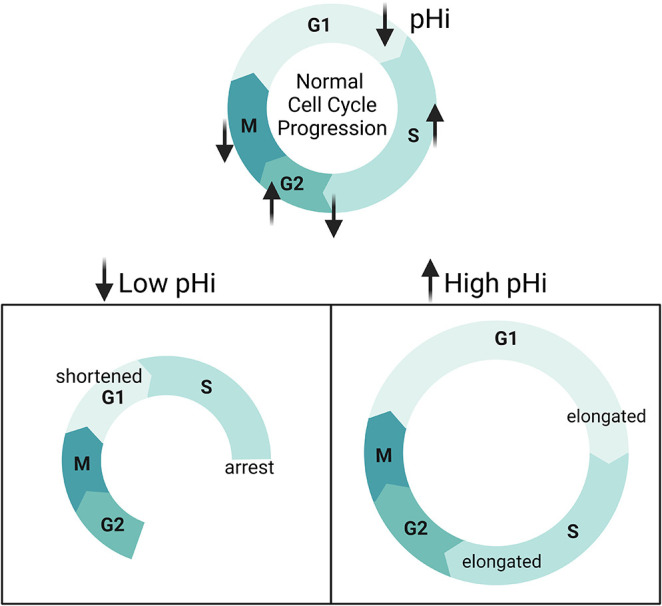
**Single-cell pHi is dynamic during cell cycle progression and regulates G1 exit, S phase duration and S/G2 transition.** During cell cycle progression, pHi decreases at the G1/S boundary, increases in mid-S phase before dropping in late S, increases through G2 and decreases in the period leading up to division. When pHi is experimentally decreased, cells have a shortened G1 and fewer S/G2 transitions. When pHi is experimentally increased, G1 and S phases are elongated. This suggests that low pHi cues G1 exit and high pH is necessary for G2 entry.

We also found that high pHi significantly elongated S phase (Fig. 7F, 16.3±2.8 h) compared to that in CRL (Fig. 7F, 12.3±3.3 h), whereas low pHi inhibited the S/G2 transition for all but 7.8% of cells (Fig. 7E). This suggests that high pHi is a requirement for the S phase transition to G2, but there is also a need for low pHi for correct timing of S phase duration. The requirement for an increase and decrease in pHi is supported by the synchronized single-cell pHi data, which showed an increase in pHi during mid-S phase and a decrease in late S phase or near the S/G2 transition (Fig. 6A). Thus, our data suggests that without an increase in pHi, cells cannot complete the S/G2 transition, but that dynamic pHi is required for correct S phase timing.

We did not measure a significant difference in G2 phase length with high pHi compared to that in CRL (Fig. 7G). Unfortunately, because low pHi cells could not complete the S/G2 transition, G2 phase times could not be measured for this treatment. M phase duration for low pHi cells could be measured only for cells in G2 or M during the start of the experiment. Based on these data, we hypothesized that if a high pHi threshold was already met during early G2, low pHi cells had the ability to complete division. This hypothesis aligns with the time-lapse data showing high pHi ∼5 h prior to division followed by a rapid acidification during mitosis (Figs 4C and 5C). We saw no significant differences in M phase timing with pH manipulation. However, the longer acquisition window (20 min) of the time-lapse experiments in this work could also reduce accuracy of M phase length measurements.

The single-cell measurements presented here, both via endpoint assays and single-cell time-lapse measurements, show novel oscillating pHi dynamics throughout the cell cycle. Our data support prior work using ion transporter knockdown that showed that high pHi regulates S phase length (Flinck et al., 2018a) and G2/M (Putney and Barber, 2003). However, our work also reveals novel decreases in pHi during G1/S, late S and mitosis (Fig. 6A). Our combined use of single-cell pHi manipulation and cell cycle reporters show that pHi plays an important role in regulating the cell cycle, particularly for correct timing of G1 exit, S phase progression, and G2 entry (Fig. 8). Taken together, these results indicate that decreased pHi might be a cue for G1 exit but prevents cells from completing S/G2 and G2/M. We also found that dynamic increases and decreases in pHi are required for S phase, and increased pHi is necessary for G2 entry. In conclusion, our work suggests that oscillating single-cell pHi not only reports on but regulates cell cycle progression in single cells.

## DISCUSSION

Intracellular pHi dynamics have been implicated in diverse cellular processes, such as differentiation (Ulmschneider et al., 2016), proliferation (Flinck et al., 2018b), migration (Martin et al., 2011) and apoptosis (Sergeeva et al., 2017). However, we have limited mechanistic understanding of how spatiotemporal and single-cell pHi dynamics regulate cell behaviors. This is partially due to reliance on population analyses, non-physiological environments, or genetic ion transporter ablation approaches to link pHi and phenotype (Czowski et al., 2020).

Here, we sought to characterize the relationship between pHi and cell cycle progression in single cells. Prior work at the population level has suggested that there is a role for pHi in regulating cell cycle progression (Flinck et al., 2018b). We show that single-cell pHi is dynamic and oscillates over an entire cell cycle, with pHi significantly decreasing at the G1/S boundary, increasing in mid-S, decreasing in late S phase, increasing through G2 and peaking at G2/M, before acidifying during mitosis and recovering in daughter cells. Here, we present three key results suggesting a regulatory link between pHi dynamics and cell cycle at both the population and single-cell levels.

First, we show that pHi significantly decreases at the G1/S boundary. These results were consistent regardless of which cell cycle synchronization method was used. The single-cell pHi manipulation data suggests that decreased pHi is a cue for G1 exit, as low pHi significantly shortened G1 and high pHi significantly elongated G1 compared to what was seen in control cells. These results indicate a novel regulatory role for pHi acidification in regulating G1 exit. Future work will investigate what molecular drivers might be responding to the low pHi observed at the G1/S transition to time G1 exit or S phase entry in single cells.

Second, we show that pHi increases in mid-S phase and decreases before the S/G2 transition. Experimentally lowering pHi inhibited S/G2 transitions and raising pHi allowed for increased success of S/G2 transitions, but we also observed significantly elongated S phase with high pHi. These data suggest that increased pHi is necessary for successful entry into G2, but dynamic pHi (both increases and decreases) are important for proper timing of S phase. These data confirm prior results at the population level showing pHi increases in S phase (Flinck et al., 2018a) and successful transition through G2/M (Putney and Barber, 2003). However, our work also reveals novel temporally regulated decreases in pHi that might be necessary for successfully timed G1/S and S/G2 transitions.

Third, we show that single-cell pHi peaks at G2/M and rapidly acidifies during M phase. The increase in pHi during G2/M confirms prior work showing that increased pHi correlates with increased G2/M transition (Putney and Barber, 2003; Sellier et al., 2006). However, our data also suggests a novel role for intracellular acidification during late M phase and division. Supporting our results, recent work investigating intracellular lactate levels during cell cycle progression found that lactate regulates the anaphase-promoting complex (APC) and is important for efficient mitotic exit (Liu et al., 2023). As lactate production releases protons as a byproduct, this recent work fits with the rapid acidification we measured in single cells during mitosis. Future work by our laboratory will apply optogenetic tools to spatiotemporally change pHi in single cells (Donahue et al., 2021) to further characterize roles for decreased pHi in regulating M phase timing or successful chromosomal segregation and division.

We also note that for most cells in the pHi manipulation experiments, we only monitored one division. Prior work on single-cell cell cycle progression has suggested that mother cell mitogen history affects daughter cell cycle (Min et al., 2020). Under E+S treatment, no daughter cells in the dataset successfully continued a second round of the cell cycle after the G1/S transition, although a handful of daughter cells divided again in the control population (∼25%). Future work will explore how dysregulated pHi dynamics in the mother cell alters or modulates daughter cell outcomes.

Prior work has suggested that increased pHi in cancer promotes proliferation and tumorigenesis (Korenchan and Flavell, 2019; White et al., 2017a). Here, we show that whereas median pHi is increased in cancer cells compared to in normal cells from the same tissue, single-cell pHi is heterogeneous and dynamic during cell cycle progression. However, the acidification of pHe also plays a role in maintaining these phenotypes (Boedtkjer and Pedersen, 2020) and altered pHe has been linked to cell cycle progression phenotypes in unicellular organisms (Aerts et al., 1985; Gillies and Deamer, 1979) as well as in mammalian cells (Deutsch et al., 1982). The two-dimensional imaging in large relative volumes of bulk medium performed here is unlikely to reflect proliferation in a tissue, and extremely unlikely to mimic the competitive and confined environment that is produced during tumor growth and compression of surrounding tissue. Thus, in order to fully recapitulate roles for pHi in proliferation of a tissue or tumor, we must explore the role for spatiotemporal pHi dynamics in three-dimensional environments. Future work will measure pHi gradients in normal and cancer cells in three dimensional environments with various extracellular matrix compositions and stiffnesses. This future work will have implications for how spatiotemporal pHi dynamics regulate biology and could lead to new therapeutic routes for limiting pHi-dependent behaviors in diseases with dysregulated pHi, such as cancer (Harguindey et al., 2017; White et al., 2017a) and neurodegeneration (Majdi et al., 2016).

Single-cell techniques can elucidate single-cell behaviors and reveal heterogeneity not found at the population level. Here, we addressed a critical need in the field to understand how pHi dynamics regulate single cells during cell cycle progression. These pHi dynamics could be essential for understanding the complex cell biology that integrates single-cell and tissue-level behaviors. For example, prior work showed pHi gradients are generated in morphogenetic tissues (Weiß and Bohrmann, 2019). Our work now supports the hypothesis that bursts of synchronized cell proliferation might underlie these observations. More work is necessary to determine how temporal pHi gradients are generated during cell cycle phase transitions and whether a threshold of pHi changes is required. With our data establishing a framework of pHi regulation during an entire cell cycle, future work will determine which pH-sensitive proteins could be mediating and correctly timing pH-dependent cell cycle progression.

## MATERIALS AND METHODS

### Cell culture and conditions

Complete medium for H1299 cells (ATCC CRL-5803) was RPMI 1640 (Corning, 10-040-CV) supplemented with 10% fetal bovine serum (FBS, Peak Serum, PS-FB2); A549 (ATCC CCL-185) and MDA-MB-231 (ATCC HTB-26) cells were cultured in DMEM (Corning, MT10013CVV) supplemented with 10% FBS; and NL20 (ATCC CRL-2503) cells in Ham's F12 (Lonza, 12001-578) supplemented with 4% FBS, 1.5 g/l sodium bicarbonate (Sigma, S6014), 2.7 g/l glucose (VWR, BDH9230), 2.0 mM Glutamax (Gibco, 35050079), 0.1 mM nonessential amino acids (Lonza, BW13114E), 0.005 mg/ml insulin (Sigma, I1882), 10 ng/ml EGF (Peprotech, AF-100-15), 1 μg/ml transferrin (BioVision, 10835-642), 0.5 μg/ml hydrocortisone (Sigma, H0888). MCF10A (ATCC CRL-10317) cells were cultured in 50% DMEM/50% F12 with GlutaMax (Invitrogen, 10565-018) supplemented with 5% horse serum (Invitrogen, 16050-122), 0.02 μg/ml EGF (Peprotech, AF-100-15), 5 μg/ml hydrocortisone (Sigma, H-0888), 0.01 mg/ml insulin (Sigma, I-1882), 0.1 μg/ml Cholera toxin (Sigma, C-8052) and 1% penicillin-streptomycin (Corning, 30-001-Cl). All cells were maintained at 5% CO_2_ and 37°C in a humidified incubator. All cell lines were authenticated and tested for mycoplasma in November 2022.

### Transfections and stable cell line selection

H1299 cells were transfected with the pCDNA3-mCherry-SEpHluorin (Koivusalo et al., 2010) (mCh-pHl) or pLenti-CMV-Blast-PIP-FUCCI using Lipofectamine 2000 (Life Technologies, 11668019) as per manufacturer's instructions. After 24 h, cells were trypsinized and plated at a low dilution in a 10 cm dish with medium containing 0.8 mg/ml geneticin (Gibco, 10131035). Cloning cylinders were used to select colonies expressing mCh-pHl for expansion. A final clone was selected based on microscopy assay for mCh-pHl expression and comparison of cell morphology and pHi to parental H1299. For H1299-FUCCI, cells were trypsinized after 24 h transfection and plated at low dilutions (50 cells/ml) in a 96-well plate in medium containing 0.8 mg/ml blasticidin (Thermo Fisher Scientific, BP264725). Wells with equal expression were further expanded and screened on a microscopy assay for FUCCI expression and for similar cell morphology and pHi compared to parental H1299.

Lentiviral transfection was used to generate stable mCh-pHl expression in NL20 and A549 cells. Production of the virus was carried out in 293FT cells (gift from Siyuan Zhang, UT Southwestern Medical Center, Dallas, TX, USA). Cells were grown to near confluency in a 10 cm dish and transfected with plx304-mCherry-SEpHluorin (gift from Yi Liu and Diane Barber at UCSF, San Francisco, USA) and two packaging plasmids psPAX2 (Addgene #12260) and pmd2.G (Addgene #12259), were gifts from Siyuan Zhang (UT Southwestern Medical Center, Dallas, TX, USA). A total of 3 µg each of the plx304-mCherry-pHluorin, psPAX2 and pmd2.G were transfected into a nearly confluent 10 cm dish of 293FT cells using Lipofectamine2000 for 18 h. Medium was changed, and cells were incubated another 3 days. Viral supernatant was collected from the cells and centrifuged for 15 min at 1811 ***g***. The supernatant was passed through a 0.2 µm polyethersulfone filter, flash-frozen in liquid nitrogen in 1 ml aliquots and stored at −80°C.

NL20 and A549 cells were plated in a six-well plate for viral transduction. After 24 h, viral supernatant was diluted 1:1.6, 1:3 and 1:10 into antibiotic-free medium (depending on cell line) with 10 µg/ml Polybrene (Sigma, TR-1003-G) and added to separate wells and incubated for 48–72 h. Transduced cells were moved to a 10 cm dish and selected with 0.8 mg/ml blasticidin. NL20 cells were plated at low density in 96-well plates (50 cells/ml). Colonies expressing mCh-pHl were expanded, and a final NL20-mCh-pHl clone was chosen with matched morphology and pHi of parentals. A549 cells were sorted using fluorescence-activated cell sorting (FACS), and a population sort according to mCherry expression was used for all imaging experiments after confirmation with microscopy.

### BCECF plate reader assays

Cells were plated at 4.0×10^5^–8.0×10^5^ cells/well in a 24-well plate and incubated overnight. Cells were treated with 2 μM 2′,7′-bis-(2-carboxyethyl)-5-(and-6)-carboxyfluorescein, acetoxymethyl ester (BCECF-AM; VWR, 89139-244) for 20 min at 37°C and 5% CO_2_. NL20 and H1299 cells were washed three times for 5 min each time with a pre-warmed (37°C) HEPES-based wash buffer (30 mM HEPES pH 7.4, 145 mM NaCl, 5 mM KCl, 10 mM glucose, 1 mM MgSO_4_, 1 mM KHPO_4_, 2 mM CaCl_2_, pH 7.4) to match their low bicarbonate medium (RPMI, Ham's F12) and A549 cells were washed three times for 5 min each time with a pre-warmed (37°C) bicarbonate-based wash buffer (25 mM HCO_3_, 115 mM NaCl, 5 mM KCl, 10 mM glucose, 1 mM MgSO_4_, 1 mM KHPO_4_, 2 mM CaCl_2_, pH 7.4) to match its high bicarbonate medium (DMEM). Two nigericin buffers (25 mM HEPES, 105 mM KCl, 1 mM MgCl_2_) were supplemented with 10 μM nigericin (Thermo Fisher Scientific, N1495), pH was adjusted to ∼6.7 and ∼7.7, and were pre-warmed to 37°C. Fluorescence was read (excitation of 440 and 490 nm, both with emission at 535 nm) on a Cytation 5 (BioTek) plate reader incubated at 37°C with 5% CO_2_. Kinetic reads were taken at 15-s intervals for 5 min, using a protocol established within BioTek Gen5 software. After the initial pHi read, the HEPES/bicarbonate wash was aspirated and replaced with one of the nigericin buffer standards, and cells were incubated at 37°C with 5% CO_2_ for 7 min. BCECF fluorescence was read by the plate reader as above. This process was repeated with the second nigericin standard. As it takes significant time to equilibrate CO_2_ in the plate reader, we did not measure nigericin standardizations without CO_2_. The mean intensity ratio (490/440 values) was derived from each read. Measurements were calculated from a nigericin linear regression using exact nigericin buffer pH to two decimal places (Grillo-Hill et al., 2014).

### Western blotting

Protein lysates were collected from 35-mm dishes or six-well plates frozen at time points matched to imaging. Ice-cold lysis buffer [50 mM Tris-HCl pH 7.5, 150 mM NaCl, 1 mM dithiothreitol (DTT), 1 mM EDTA, 1% Triton X-100 and protease inhibitor cocktail (Roche)] was added to the samples and incubated for 15 min on ice. Cells were scraped and centrifuged for 10 min at 13,000 ***g*** at 4°C. The supernatant was retained, and protein concentration was determined by Pierce™ BCA (Thermo Fisher Scientific, 23225) protein assay.

A total of 15 μg protein was loaded onto an SDS-PAGE gel that was run for 3 h at 120 V in 1× Tris-glycine (3.02 g/l Tris, 14.4 g/l glycine, 1.0 g/l SDS). Either a wet-transfer system or a Trans-Blot Turbo Transfer System (Bio-Rad) was used to transfer the proteins to a PDVF membrane (pre-wet with methanol). For the wet transfer, 1× transfer buffer (141 g/l glycine and 0.3 g/l Tris base) with 20% methanol for 1.5 h at 100 V was used. For the Trans-Blot Turbo Transfer, Bio-Rad transfer buffer was used according to the manufacturer's protocol (7 min). Membranes were blocked in 5% BSA in TBST (2.42 g/l Tris, 8 g/l NaCl and 0.1% Tween 20) for 2 h then divided for blotting. Primary antibodies used were against: cyclin A2 (1:500; Abcam, ab38), cyclin B1 (1:1000; Cell Signaling, 12231), cyclin E1 (1:1000; Cell Signaling, 4129) and actin (1:1000; Santa Cruz Biotechnology, 2Q1055). Membranes were incubated with primary antibody solution overnight at 4°C with shaking (4 h at room temperature with shaking for actin). Membranes were washed three times for 10 min each time with TBST at room temperature with shaking and incubated with secondary antibodies [1:10,000; goat anti-mouse-IgG conjugated to HRP (Bio-Rad, 1721011) or goat anti-rabbit-IgG conjugated to HRP (Bio-Rad, 1706515)] for 2 h at room temperature with shaking. Membranes were washed 3×10 min TBST at room temperature with shaking, developed using SuperSignal™ West Pico PLUS Chemiluminescent Substrate (Thermo Fisher Scientific, 34578), and visualized using a ChemiDoc MP Imaging System (BioRad). ImageJ was used for protein quantification, normalized to loading control.

### Double-thymidine block

Cells were plated at 10% confluency in five replicate 35-mm glass-bottomed dishes and five replicate 6-well plates (for protein lysate collection) and incubated overnight. Dishes were identically treated with 2 mM thymidine (Sigma, T9250) for 18 h, washed with Dulbecco's phosphate-buffered saline (DPBS) quickly (<30 s), and incubated with fresh complete medium for 9 h, then treated for another 18 h with 2 mM thymidine. Cells were released with a quick (<30 s) DPBS wash and replaced with fresh complete medium. Imaging of the 0 h time point was initiated 20 min after release. Subsequent images were collected at 4, 8, 12 and 24 h after release in respective medium. Matched dishes at each time point were washed twice with DPBS and frozen at −80°C for protein lysate collection and immunoblot analysis of cyclins.

For time-lapse imaging, the double-thymidine block was used as explained above on a single 35-mm glass-bottomed dish supplemented with 1% penicillin and streptomycin (Corning, 30-001-C1) to avoid bacterial contamination during time-lapse microscopy. Hoechst 33342 solution (Thermo Fisher Scientific, 62249) was added to the cells (1:20,000) before release and incubated for 15 min. Dye and thymidine were removed, and cells were washed with DPBS to release cells. Fresh medium was added, and images were collected every 20 min for 24 h. Acquisition parameters were: 700 ms exposure time and 8% laser power for GFP; 700 ms exposure time and 10% laser power for TxRed; and 100 ms exposure time and 5% laser power DAPI. A single *Z*-plane was collected to avoid photobleaching. Nigericin standards were carried out as previously described (Grillo-Hill et al., 2014). For the asynchronous time-lapses, cells were plated the day prior to imaging and images were collected identically to thymidine-treated cells.

### Palbociclib synchronization

Cells were plated at 10% confluency in five replicate 35-mm glass-bottomed dishes and five replicate six-well plates (for protein lysate collection) and incubated overnight. Dishes were identically treated with 0.1 µM palbociclib (PD-0332991) (Selleck, S1116) for 24 h. Cells were washed with DPBS quickly (<30 s) and released with complete fresh medium. Imaging of the 0 h time point was initiated 20 min after release. Subsequent images were collected at 4, 8, 12, 24 and 36 h post-release in respective medium (three replicates collected for 0–24 h time points, and two replicates where a 36 h time point was also collected). Matched dishes at each time point were washed twice with DPBS and frozen at −80°C for protein lysate collection and immunoblot analysis of cyclins.

### FUCCI cell cycle assays

For H1299-FUCCI time-lapses, cells were plated in a four-well imaging dish (10,000 cells/well) and supplemented with 1% penicillin and streptomycin (Corning, 30-001-C1) to avoid bacterial contamination during long-term acquisition. For HCO_3_^−^ supplementation, 35-mm glass-bottomed dishes were used. Hoechst 33342 dye was added to the cells (1:20,000) at 2–4 h prior to imaging and incubated for 15 min; then, dye solution was removed, and fresh medium was added to the cells. Experiments were started immediately after treatments were added to the cultured medium and images were collected every 20 min for 36 h. Optimal acquisition parameters were as follows: 200 ms exposure time and 8% laser power for GFP; 800 ms exposure time and 10% laser power for mCherry; and 200 ms exposure time and 5% laser power DAPI.

### Microscopy

The imaging protocol was derived from Grillo-Hill et al. (2014). Cells were plated on a 35-mm imaging dish with a 14-mm glass coverslip (Matsunami, D35-14-1.5-U) a day before imaging. Microscope objectives were preheated to 37°C, and the stage-top incubator was preheated to 37°C and kept at 5% CO_2_/95% air. Confocal images were collected on a Nikon Ti-2 spinning disk confocal with a 40× (CFI PLAN FLUOR NA 1.3) oil immersion objective. The microscope is equipped with a stage-top incubator (Tokai Hit), a Yokogawa spinning disk confocal head (CSU-X1), four laser lines (405 nm, 488 nm, 561 nm and 647 nm), a Ti2-S-SE motorized stage, multi-point perfect focus system and an Orca Flash 4.0 CMOS camera. Hoechst 33342 dye (405 nm laser excitation, 455/50 nm emission), pHluorin (488 nm laser excitation, 525/36 nm emission), TxRed (561 nm laser excitation, 605/52 nm emission), mCherry (561 nm laser excitation, 630/75 nm emission) and SNARF (561 nm laser excitation, 705/72 nm emission) were used. Acquisition times for each fluorescence acquisition ranged from 100 to 800 ms.

### Immunofluorescence assays

Cells were plated in a four-well imaging dish (20,000 cells/well) overnight, then treated with pHi manipulation medium or etoposide (positive control to validate the anti-γ-H2AX antibody, 10 μM) for 24 h. Cells were rinsed with DPBS and fixed in 3.7% formaldehyde (Alfa Aestar, 33314) at room temperature for 10 min. Cells were washed three times for 2 min each time with DPBS, then incubated lysing buffer (0.1% Triton-X in DPBS) for 10 min at room temperature. Cells were washed three times for 2 min each time with DPBS, then incubated in blocking buffer (1.0% BSA in DPBS) for 1 h at room temperature with rocking followed by three 2 min washes in DPBS. Cells were then incubated overnight at 4°C with anti-γ-H2AX antibody (1:100; Cell Signaling, 9718S) in antibody buffer (0.1% Triton-X and 1.0% BSA in DPBS). Cells were washed three times for 2 min each time in DPBS, then incubated with secondary antibody (Alexa Fluor 488-conjugated goat anti-rabbit-IgG, 1:1000, Invitrogen, A11008) for 1 h at room temperature. After three 2 min washes in DPBS, Hoechst 33342 dye (1:20,000) in antibody buffer was added for 15 min, then removed. Cells were imaged in DPBS on the spinning disk confocal microscope as above (Hoechst dye, 405 nm laser excitation, 455/50 nm emission; Alexa Fluor 488, 488 nm laser excitation, 525/36 emission).

### SNARF microscopy assays

We note that we were not able to isolate an MCF10A cell line stably expressing mCherry–pHluorin, so comparisons between MCF10A and MDA-MB-231 were performed using the pH-sensitive dye SNARF [4-(and-6)-carboxySNARF-1 acetoxymethyl ester, acetate; Invitrogen, C1272]. Cells were plated at 4.0×10^5^ cells/well in an imaging dish (Matsunami). Conditioned medium was removed, and cells were treated with 20 μM SNARF in serum-free medium for 15 min and then medium was replaced with conditioned medium. Images were collected similarly to those for mCh-pHl experiments using nigericin standardization.

### Intracellular pH imaging and data collection

For all pHi imaging (SNARF and mCherry–pHluorin), initial fields of view (FOVs) were collected on the cells in their respective media. For all imaging, nigericin buffers were prepared identically to as for the BCECF assays, and all buffer exchanges were carried out on the stage incubator to preserve *XY* positioning. Multiple *Z*-planes were collected with the center focal plane maintained using the Nikon Ti2 Perfect Focus System (PFS).

For time-lapse pHi and FUCCI imaging, a single *Z*-plane was collected to avoid excess light, and additional water was added to the stage top incubator at 18 h. For pHi manipulation validation, cells were plated at 20% confluency on a 35-mm imaging dish with a 14-mm glass coverslip and incubated overnight. For Fig. 6, cells were treated with a combination of 15 μM EIPA and 30 μM S0859 (E+S), 1 µM concanamycin A (CMA), 100 mM NaHCO_3_^−^ (HCO_3_^−^) or 20 mM ammonium chloride (NH_4_Cl) diluted in fresh medium and incubated for 24 h. For Fig. S8, cells were treated with E+S or NH_4_Cl for 4, 8 or 12 h. Both imaging collection and pHi calculations were completed identically to those for other single-cell pHi measurement experiments. E+S-, HCO_3_^−^- and NH_4_Cl-treated cells with respective controls were corrected for photobleaching by collecting images of cells in nigericin buffers (pH 7.4) with treatment supplemented but without nigericin present.

### Image quantification

Images were background-subtracted using a region of interest (ROI) drawn on a glass coverslip (determined by DIC). For pHi quantification, individual ROIs were drawn for each cell in each condition (initial, high pH nigericin and low pH nigericin). For SNARF assays, mean TxRed and SNARF pixel intensities were quantified for each cell and SNARF/TxRed ratios were calculated in Excel software. For mCherry–pHluorin assays, mCherry aggregates were removed using thresholding holes and then pHluorin and mCherry pixel intensities were quantified for each cell. The pHluorin/mCherry ratios were calculated in Excel software. In both cases, a cutoff of 100 arbitrary units (a.u.) was used for both pHluorin and mCherry intensity values after exporting. For each cell, nigericin values were used to generate a standard curve, and pHi was back-calculated from the single-cell standard curve.

For FUCCI analysis, cells were tracked using NIS Elements Advanced Research Software analysis software (Nikon) and nuclear ROIs based on DNA stain. In case of improper tracking, manual tracking was used to redraw ROIs. Manual tracking was also used during mitosis when the signals diffused throughout the cell. mVenus and mCherry intensities were exported from matched single-cell nuclear ROI at each time point over 36 h. Cell cycle phases were determined by mVenus or mCherry fluorescence intensity, adapted from Grant et al. (2018). For each individual cell trace, including subsequent daughter cells, an Excel macro was used to determine time points for mVenus and mCherry cutoffs. G1/S was defined as a decrease in mVenus signal below 5% of maximum mVenus intensity. As validation of G1/S, S phase entry was defined as the first time point after an mCherry minimum that showed a 3% (determined from mCherry maximum) increase in mCherry intensity compared to the previous point. S/G2 was defined as point at which mVenus intensity rose above 2% of the mVenus maximum compared to the previous point. G2/M and M/G1 were defined by nuclear envelope breakdown and division into two daughter cells, respectively.

For γ-H2AX staining, after background subtraction, the nuclear ROIs were drawn based on DNA stain and GFP intensities were exported for each treatment condition.

### Statistics

GraphPad Prism was used to prepare graphs and perform statistical analyses. Normality tests were performed on all data sets as well as outlier test using the ROUT method (Q=1%). For normally distributed data, an unpaired two-tailed *t*-test (Fig. 5G; Fig. S1A–C) or paired two-tailed *t*-test (Fig. 2F–H; Fig. 3F–H) was used. A Mann–Whitney test was used for non-normal unpaired data (Figs 1F,G, 6B, 7E–H; Fig. S3C, S4C, S5F, S8). For time-lapse data (Figs 4C,F, 5C,F), a one-sample Wilcoxon test was used, compared to a theoretical mean of 0. For non-normal unpaired data with more than two sets, a Kruskal–Wallis test with Dunn's multiple comparisons test was used (Figs 2D,I, 3D,I; Figs S3A, S4A, S5C,E). Values were binned at 0.02 in all frequency distributions. All significance was indicated in figures by the following: *P<0.05; **P<0.01; ***P<0.001.

## Supplementary Material

10.1242/joces.260458_sup1 Supplementary information
